# Toward a Literature-Driven Definition of Big Data in Healthcare

**DOI:** 10.1155/2015/639021

**Published:** 2015-06-02

**Authors:** Emilie Baro, Samuel Degoul, Régis Beuscart, Emmanuel Chazard

**Affiliations:** Department of Public Health, EA 2694, University of Lille, 1 Place de Verdun, 59045 Lille Cedex, France

## Abstract

*Objective.* The aim of this study was to provide a definition of big data in healthcare. *Methods.* A systematic search of PubMed literature published until May 9, 2014, was conducted. We noted the number of statistical individuals (*n*) and the number of variables (*p*) for all papers describing a dataset. These papers were classified into fields of study. Characteristics attributed to big data by authors were also considered. Based on this analysis, a definition of big data was proposed. *Results.* A total of 196 papers were included. Big data can be defined as datasets with Log⁡(*n*∗*p*) ≥ 7. Properties of big data are its great variety and high velocity. Big data raises challenges on veracity, on all aspects of the workflow, on extracting meaningful information, and on sharing information. Big data requires new computational methods that optimize data management. Related concepts are data reuse, false knowledge discovery, and privacy issues. *Conclusion.* Big data is defined by volume. Big data should not be confused with data reuse: data can be big without being reused for another purpose, for example, in omics. Inversely, data can be reused without being necessarily big, for example, secondary use of Electronic Medical Records (EMR) data.

## 1. Introduction

The 21st century is an era of big data involving all aspects of human life, including biology and medicine [1]. With the advance in genomics, proteomics, metabolomics, and other types of omics technologies during the past decades, a tremendous amount of data related to molecular biology has been produced [2]. In addition, the transition from paper medical records to EHR systems has led to an exponential growth of data [3]. As a result, big data provides a wonderful opportunity for physicians, epidemiologists, and health policy experts to make data-driven decisions that will ultimately improve patient care [3]. As Margolis stated, “Big data are not only a new reality for the biomedical scientist, but an imperative that must be understood and used effectively in the quest for new knowledge” [4].

To date, however, the term “big data” does not have a proper definition in the MeSH (Medical Subject Headings) database yet. A precise, well-formed, and unambiguous definition is a requirement for a shared understanding of the term big data. The objective of this work is to provide a definition of big data in healthcare through a review of the literature.

## 2. Material and Methods

### 2.1. Search Strategy

For this literature review, we conducted a systematic search of the PubMed database for all papers published until May 9, 2014, using the keywords “big data.” To be fully inclusive, we did not define a start date. We used the following PubMed query:

- (big data[Title/Abstract]) AND (“1900/01/01”[Date - Publication]: “2014/05/09”[Date - Publication]).

Titles and abstracts were reviewed by a human for eligibility. Papers were excluded if they were not directly related to healthcare or if big data was not found to be the topic of the paper.

We then attempted to retrieve the full-text papers. We used online search facilities (the Free PMC database, Google, and Google Scholar), resources, and services of the Lille University library and tried to directly contact the first or corresponding author. Full-text papers were then read.

Each of the remaining papers was included in the analysis and classified either as a paper describing a dataset, a dissertation, or a review of the literature.

### 2.2. Data Collection Process

For each paper, we collected the following information: title, year of publication, journal title, specialty area, type of paper (paper using a dataset, dissertation, and literature review), the field of study, and characteristics given by authors to big data and to data reuse. In case the paper dealt with a dataset, we also collected the number of statistical individuals (*n*) and the number of variables (*p*). It should be noted that the number of statistical individuals *n* is not necessarily physical persons but can also be, for example, gene sequences. The number of variables *p* could be, for example, the number of physicochemical properties used to classify amino acids [5], the performance metrics adopted to evaluate model performance [6], or the number of features of medical claims. In this last case, the number of individuals *n* is represented by the number of records of medical claims [7].

### 2.3. Analysis and Classification

Statistical analyses were performed with R statistical computing software [8]. In this paper, the notation “Log” denotes the decimal (or common, or decadic) logarithm, and the notation “CI_95_” denotes 95% confidence intervals. CI_95_ of binary variables were computed using the binomial law.

#### 2.3.1. Time Evolution of Publication about Big Data in Healthcare

To analyze the evolution of publication in healthcare, we draw a graph showing the annual publication of papers included in our review and a graph showing the annual publication of papers which were describing a dataset. We also noted the number of journals which published papers about big data in healthcare per year.

#### 2.3.2. Time Evolution of the Size of Big Data in Healthcare

In order to see the evolution of what authors refer to as “big data,” from papers describing a dataset, we plotted the decimal logarithm of the product of the number of statistical individuals (*n*) and the number of variables (*p*),  Log⁡(*n*∗*p*), as a function of the year.

#### 2.3.3. Number of Individuals and Variables in Each Field of Study

The numbers *n* and *p* were analyzed with respect to the field of study. To this end, the probability density functions of Log(*n*),  Log(*p*), and Log(*n*∗*p*) were plotted with respect to fields of study. Finally, Log(*p*) as a function of Log(*n*) was plotted with respect to fields of study.

### 2.4. Characteristics of Big Data

Characteristics attributed to big data by the authors in free text were noted as reading all the papers included in the analysis and were then sorted out by categories.

### 2.5. Proposal of a Definition of Big Data

We then gathered to propose a definition of big data in healthcare. A difference was made between definition, properties, and related concepts. A dataset that matches the definition qualifies as “big data,” and thus has the properties that are proposed. Conversely, a dataset that has some or all of the listed properties does not necessarily qualify as “big data.” Finally, related concepts refer to properties that are not systematically related to big data.

We attempted to bring out a threshold of the volume of big data on the basis of findings from this literature review. The threshold resulted from a discussion between the authors of this paper, taking into account sizes of actual datasets, but also properties that are attributed to big data by the authors of the papers included in this literature review.

## 3. Results

### 3.1. Search Strategy

**Figure 1:**
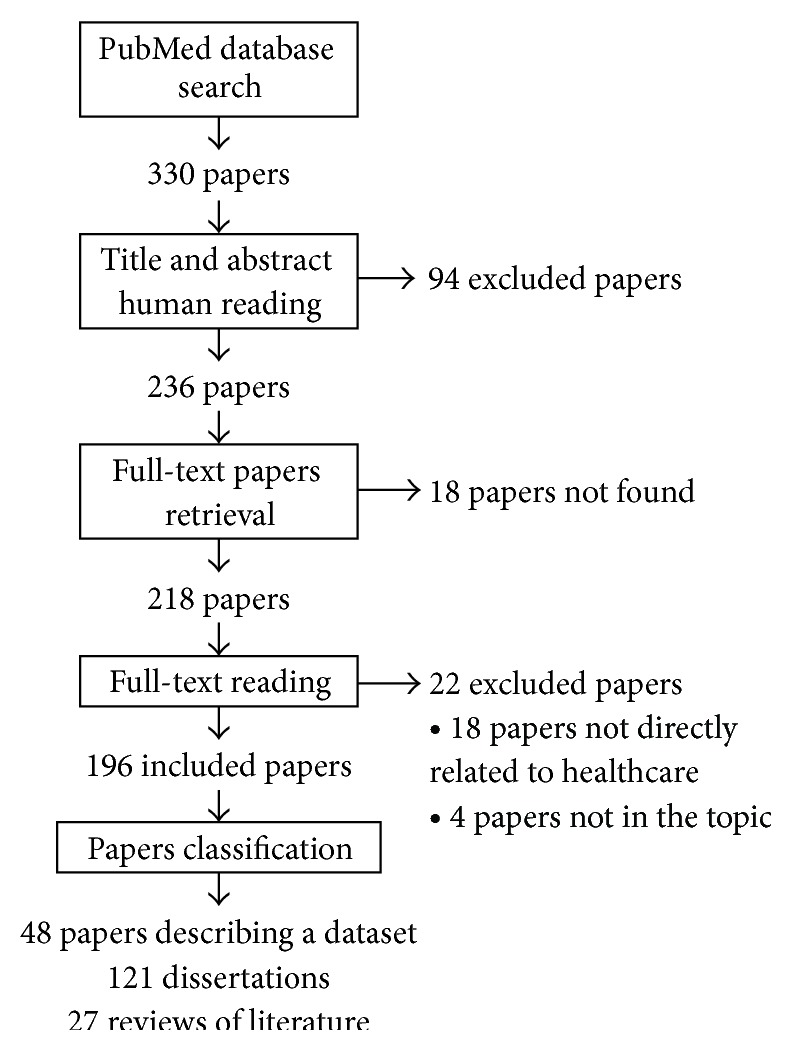
Flowchart of the literature review.

The search query yielded 330 papers. After reading titles and abstracts, 94 papers were excluded. A total of 236 papers were included for full-text review. Eighteen papers were unavailable. The full-texts of the remaining 218 papers were read. After applying the exclusion criteria, 22 papers were excluded, leaving 196 papers. Papers were excluded due to the following reasons: papers not directly related to healthcare (18 papers) and papers in which big data was not the topic of the paper (4 papers). Of the 196 papers left for inclusion, there were 48 papers describing a dataset, 121 dissertations, and 27 reviews of the literature.  Figure 1 shows a detailed description of the search strategy and results.

### 3.2. Data Collection Process

The number of papers by field of study among the 48 papers describing a dataset is listed in Table 1.

Among the 48 papers describing a dataset, three main categories of studies were identified: omics, medical specialties, and public health. The term “omics” refers to biology fields of study ending in -omics, such as genomics, metabolomics, or proteomics. The main area represented is omics: 23 papers (48%, CI_95_ = [33; 63]). It is followed by medical specialties (endocrinology, infectology, immunology, neurology, and imaging): 15 papers (31%, CI_95_ = [19; 46]) and public health (bioinformatics, Electronic Health Records (EHR), epidemiology, pharmacovigilance, and public health): 10 papers (21%, CI_95_ = [10; 35]).

### 3.3. Analysis and Classification

#### 3.3.1. Time Evolution of Publication about Big Data in Healthcare

**Figure 2:**
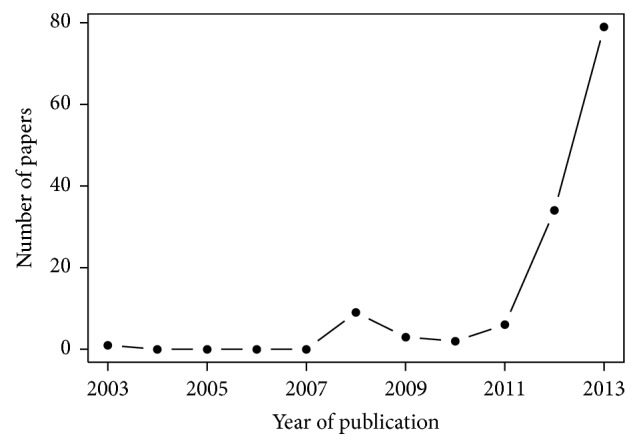
Number of papers about big data in healthcare published per year (full years only).

**Figure 3:**
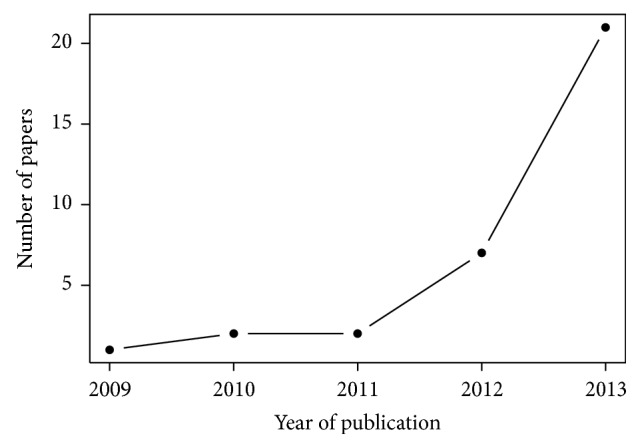
Number of papers about big data in healthcare describing a dataset per year (full years only).

Figure 2 shows the evolution of the publication of papers about big data in healthcare from 2003 to 2013. Annual publication of papers about big data in healthcare increased from 1 in 2003 to 79 in 2013. In the same way, an increase in the annual publication of papers describing a dataset can be observed (Figure 3). The 196 papers included in our review were published in 134 different journals. Among these journals, one journal published papers about big data in healthcare in 2008. There were 68 in 2013.

#### 3.3.2. Time Evolution of the Size of Big Data in Healthcare

**Figure 4:**
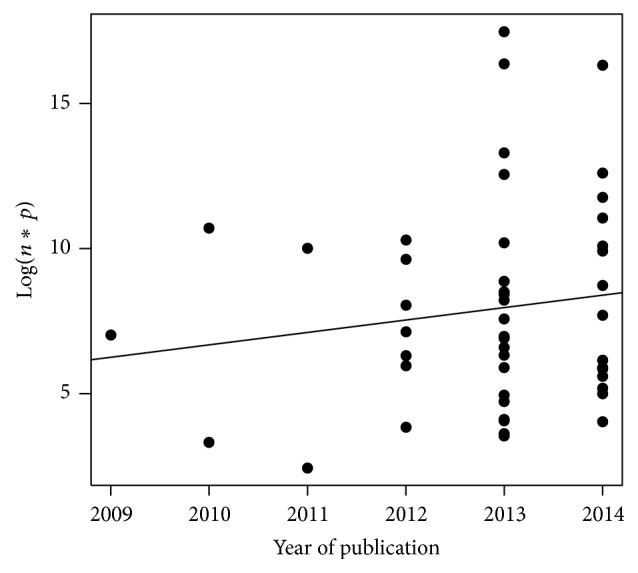
Log(*n*∗*p*) per year of publication. The continuous line represents the linear regression (*P* = 0.34).

Figure 4 illustrates the decimal logarithm of the number of statistical individuals multiplied by the number of variables (Log(*n*∗*p*)) for each year of publication of the papers that describe a dataset. We observe a nonsignificant increase of 0.43 per year (*P* value = 0.34).

#### 3.3.3. Number of Individuals and Variables in Each Field of Study

**Figure 5:**
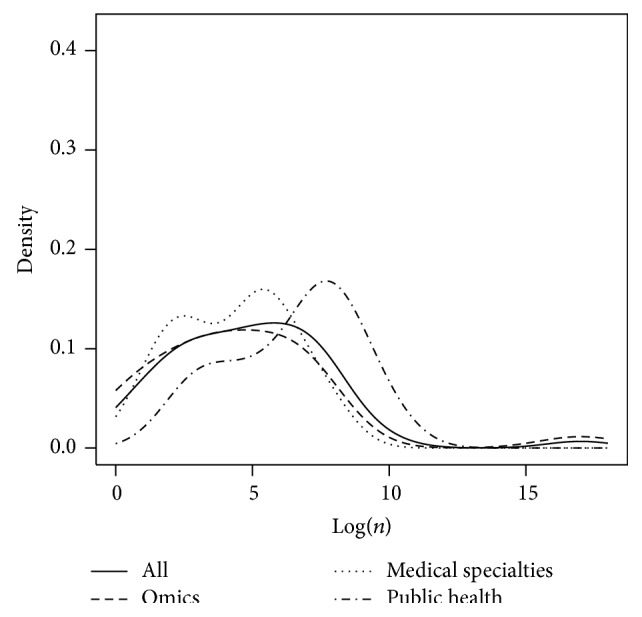
Representation of the probability density function of Log(*n*) for omics, medical specialties, public health, and all fields together.

**Figure 6:**
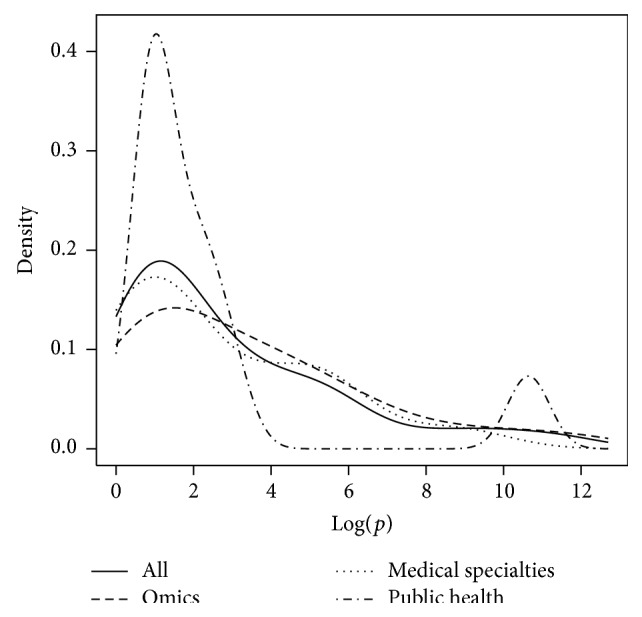
Representation of the probability density function of Log(*p*) for omics, medical specialties, public health, and all fields together.

**Figure 7:**
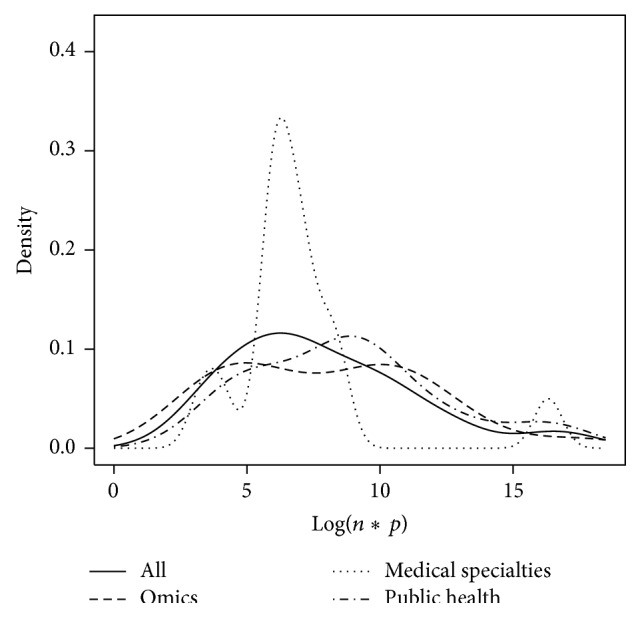
Representation of the probability density function of Log(*n*∗*p*) for omics, medical specialties, public health, and all fields together.

Figures 5, 6, and 7 represent the probability density function of Log(*n*), Log(*p*), and Log(*n*∗*p*), respectively, for omics, medical specialties, public health, and all papers. It can be pointed out that Log(*n*∗*p*) is inferior to 7 in 23 studies out of 48 (48%, CI_95_ = [33; 63]).

**Figure 8:**
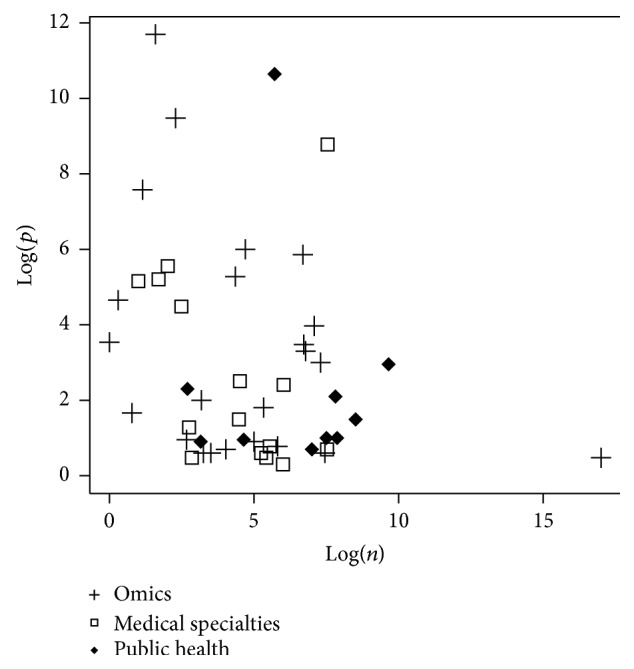
Log(*p*) as a function of Log(*n*) for omics, medical specialties, and public health. Each pictogram stands for one paper.

Figure 8 shows Log(*p*) as a function of Log(*n*) for omics, medical specialties, and public health. This figure suggests the following differences between omics, medical specialties, and public health categories:

- big data in omics concern massive data collected on a limited number of individuals: small *n*, high *p*;
- public health studies concern an important number of individuals and a low number of variables: high *n*, small *p*;
- medical specialties are characterized by an important number of individuals and variables: high *n*, high *p*.

### 3.4. Characteristics of Big Data

The main characteristic about big data found in the papers is its massive size and complexity [7, 9–17]. Big data concern “not only the sheer scale and breadth of the new data sets but also their increasing complexity” [15]. Widely used notions to describe the complexity of big data are the three “Vs”: volume, variety, and velocity [7, 18–25]. “Big Data is a term used to describe information assemblages that make conventional data, or database, processing problematic due to any combination of their size (volume), frequency of update (velocity), or diversity (variety)” [18]. Veracity is a fourth “V” sometimes added to describe big data challenge [17, 23, 26–28]. Some authors mention a fifth “V”: valorization [26, 29].

#### 3.4.1. Volume

Volume is the main characteristic mentioned by authors [7, 12, 16, 21, 23, 26, 30, 31]. “These correspond to the well-accepted notions of volume (breadth and/or depth) (…) recognized as the hallmarks of big data” [21]. “For volume, this translates today into terabytes (10^12^ bytes), petabytes (10^15^ bytes) or exabytes (10^18^ bytes)” [7]. “Volume - much greater amounts of rapidly multiplying data than were ever previously available” [25]. Some authors mention a big data threshold without clearly defining it [7, 32]: “How big is ‘Big'? (…) size is a relative term when it comes to data” [32]. “Those data are unquestionably ‘big' (order 10^17^)” [21]. Data sets used “in epidemiology (…) in fact barely pass the ‘big data' threshold” [7].

#### 3.4.2. Variety

Variety is another important characteristic of big data [7, 25, 26, 30, 31, 33–35]. Indeed, big data comes from various sources [23, 36]. Variety translates into “aggregation of widely disparate sources of data or mash-ups of data derived from independent sources” [7]. Unstructured data, for example, free text data [7, 12, 37] and images [32, 38–40], are particularly a big challenge. In healthcare, “data take many forms including numbers, text, coded data, graphics, images, physiological measures (signals), and sound. Healthcare professionals rely on all their senses, including smell, to collect assessment data from individuals” [12]. In this area, “unstructured data is expected to exponentially outpace structured data” [34]. “Electronic Medical Records (EMR) generate massive data sets, offering the challenge of how to convert largely unstructured by-products of healthcare delivery into useful assets for patients' insight” [41]. Big data “can deviate from traditional structured data (organized in rows and columns) and can be represented as semi-structured data such as XML, or unstructured data including flat files which are not compliant with traditional database methods” [33]. These data are “unstructured for analysis using conventional relational database techniques” [31].

Moreover, big data can be “volatile, that is, changing, and available only for a limited amount of time” [23].

#### 3.4.3. Velocity

Accelerated increase of data is another attribute of big data [7, 21, 23, 25, 26, 31, 42]. It is “data at or near real-time” [25]. “Velocity refers to the enormous frequency with which today's data is generated, delivered, and processed” [31].

#### 3.4.4. Challenge on Veracity

Veracity comes next: big data can be difficult to validate [17, 26–28]. “Big data must be interpreted with caution, and in context, if it is to be clinically useful” [27]. It has a low veracity. Big data can never “be 100% accurate” [28].

#### 3.4.5. Challenges on All Aspects of the Workflow

Big data raises challenges on all aspects of the workflow: from amassing [32], capturing [7, 37, 43–45], collecting [20, 46], storing [7, 20, 32, 43, 44, 47–53], data management [20, 43, 45, 54, 55], processing [9, 12, 19, 26, 47, 48, 51, 52, 56, 57], and analyzing [7, 20, 31–33, 39, 43–45, 49–55, 58–60], to peer-reviewed publications of results [45]. Big data “creates difficulties in data capture, storage, cleaning, analytics, visualization and sharing” [43]. Big data is also difficult to valorize [26, 29]: big data “is not merely large in volume; it also moves rapidly, is difficult to validate and valorize” [26].

#### 3.4.6. Challenges on Statistical and Computational Methods

Finding new statistical and computational methods is another challenge raised by big data [33, 43, 50, 51, 59, 61, 62]. Big data requires “a change of perspective, infrastructure, and methods for data collection and analyses” [62]. Visualization methods that allow us to understand the data need to be created [32, 43, 44, 57]. To make sense of big data, “the further creation of new tools and services for data discovery, integration, analysis, and visualization” [32] will be required.

#### 3.4.7. Challenges on Extracting Meaningful Information

Several authors emphasize the fact that it is necessary to derive useful information of these data [30, 44, 63, 64] and raise the question of how the data could be meaningfully interpreted: big data creates “challenges around how to meaningfully interpret the data - much of it not described using consistent standards or metadata - into information and recommendations while eliminating noise and erroneous data” [19].

#### 3.4.8. Challenges on Facilitating Information Access and Sharing

Many authors highlight the necessity of identifying ways to facilitate information access and sharing [7, 15, 30, 34, 43–46, 49, 50, 53, 62, 63, 65–67]. It is necessary to promote “collaboration among scientists” [46]. Data must be made more readily available from more open sources to better compare data.

#### 3.4.9. Not Enough Human Experts

Some authors mention the fact that the number of available human experts who have both clinical and analytic knowledge is not sufficient yet [30, 68]: “the role needs some sort of hybrid person that has clinical knowledge and analytic knowledge. We are experiencing a drought in terms of analytic experience. We don't have enough of those people in place yet” [30].

#### 3.4.10. Data Reuse

Some authors mention the fact that big data can be data that are commonly collected without an immediate use: “Massive amounts of data are commonly collected without an immediate business case, but simply because it is affordable. This data, so it is hoped, will later answer questions, most of which yet have to arise” [20]. They put into light the fact that big data are often a secondary use of data, which we can call data reuse [14, 20, 21, 41, 65, 69–72].

#### 3.4.11. False Knowledge Discovery

Some authors highlight the fact that deriving knowledge from big data can lead to false results and to conclusions that are wrong [73–75]: “Exploratory results emerging from Big Data are no less likely to be false” [75]. We cannot extract knowledge from big data without knowing the context in which data sets were collected: “big size is not enough for credible epidemiology” [74].

#### 3.4.12. Privacy Issues

One concern mentioned by several authors is privacy issues: “the increasing ease with which data may be used and reused has increased concerns about privacy and informed consent” [76]. The ability “to protect individual privacy in the era of big data has become limited” [39]. Even if large databases use pseudonymised personal confidential data that have been anonymised, they retain a residual risk of reidentification. Indeed, the identity of individuals can be determined by manipulating databases through data linkage techniques [28, 39, 66, 77]. The data torrent poses ethical challenges [15]. “The widespread implementation of EHRs and the need to share data to measure quality and manage accountable care organizations (ACOs) brings to light all of the privacy issues surrounding sharing patient data” [66]. “The ability to derive DNA-based information from non-DNA-based sources generalizes the issue of data de-identification beyond the area of genotypic data privacy and has thus potentially important consequences for privacy rules in scientific research” [39].

### 3.5. Proposal of a Definition of Big Data

A definition of big data was established on the basis of findings from the literature review. We consider that big data should exclusively be defined by volume, and we propose that a dataset could be qualified as “big dataset” only if Log(*n*∗*p*) is superior or equal to 7.

Properties of big data can be listed as follows:

- great variety,
- high velocity,
- challenge on veracity,
- challenge on all aspects of the workflow,
- challenge on computational methods,
- challenge on extracting meaningful information,
- challenge on sharing data,
- challenge on finding human experts.

Related concepts of big data are as follows:

- data reuse,
- false knowledge discovery,
- privacy issues.

The definition of big data is summed up in Table 2.

## 4. Discussion

In this work, through a detailed literature review, we tried to provide a current and quantitative definition of big data. We performed a literature review of 196 papers published until May 2014. Finally, we proposed a definition of big data in healthcare.

This systematic search should ensure that we accumulate a relatively complete census of relevant literature of big data in healthcare. However, we may have missed papers that do use big data in the research but were not included in our query because the term was not mentioned in the abstract or keywords of the paper. Those papers could be less and less frequent in the future.

Nevertheless, as there is no definition of big data, the literature can itself be wrong. It is a limitation of this inductive approach: we use observations to build a definition. The problem of defining a threshold illustrates this difficulty: the threshold of 10^7^ may appear in disagreement with the results of Figure 7. This definition of big data is simply the result of a discussion between the authors of this literature review. It has been decided based on the results of the number of individuals and of variables found in the studies describing a dataset, but it has also taken into account the characteristics of big data mentioned by the authors of all the papers included in this literature review. Thus, for example, we can consider that the problems related to computational methods do not exist for Log(*n*∗*p*) inferior to 7, even when the analysis is performed with a simple spreadsheet instead of statistical software calling for high computational capacities. However, this proposal suggests that half of the studies describing a dataset in this literature review wrongly call their dataset big data. As everyone talks about the challenges of computing and data processing, considering what we know today in practice about software and computers, it would have been difficult to admit a threshold of Log(*n*∗*p*) superior or equal to 6 (although such a threshold already excludes 35% of the studies of our review), because we know that, nowadays, such size of data is easy to deal with.

It should also be pointed out that there is an undeniable current trend of big data, which leads to the fact that the term “big data” is now used to qualify datasets that, in the past, would not have been called this way. Moreover, we can consider that the size of datasets that qualify as big data may keep on increasing due to the main property of big data, which is the challenge on data processing and the fact that computational infrastructure that is required to process these large-scale datasets may progress with time.

Data reuse has been defined as a related concept of big data because we think that there might be some confusion between these two terms: data reuse is the fact of using for decisional purposes data that were collected routinely for transactional purposes, whereas big data is related to the size of the data collection. Indeed, data can be big without being reused for another purpose: this is the case of omics, for example. Inversely, data can be reused without being necessarily big, such as secondary use of data from Electronic Medical Records (EMR).

Big data presents many opportunities for translational studies, and informatics will be the key for successful translational research [78]. As Shah stated, “translational informatics is ready to revolutionize human health and healthcare using large-scale measurements on individuals. Data-centric approaches that compute on massive amounts of data to discover patterns and to make clinically relevant predictions will gain adoption” [79]. Cloud computing could be an enabling tool to facilitate translational bioinformatics research [67].

Informatics is needed to fully harness the potential of health data and new tools are emerging to translate health data into knowledge for improved healthcare.

## Figures and Tables

**Table 1:** Number of papers by field of study among the 48 papers describing a dataset.

Field of study	Number of papers
Omics	
Genomics	18
Metabolomics	1
Proteomics	4
Medical specialties	
Endocrinology	2
Imaging	3
Immunology	1
Infectiology	1
Neurology	8
Pharmacovigilance	1
Public health	
Bioinformatics	3
EHR^*^	1
Epidemiology	2
Public health	3

^*^EHR: Electronic Health Records.

**Table 2:** Definition of big data in healthcare.

Definition	Volume: Log⁡(*n*∗*p*) ≥ 7
Properties	Great variety
	High velocity
	Challenge on veracity
	Challenge on all aspects of the workflow
	Challenge on computational methods
	Challenge on extracting meaningful information
	Challenge on sharing data
	Challenge on finding human experts

Related concepts	Data reuse
	False knowledge discovery
	Privacy issues
